# Public Hot Baths in China

**Published:** 1889-12

**Authors:** 


### PUBLIC HOT BATHS IN CHINA.

The following is a brief account of the Chinese baths, taken from personal observation. The baths being all of the same general character, it will suffice for me to describe one, situated in the town of Shanghai. There are two outer rooms, used for undressing and dressing ; the first and larger is for the poorer classes, the second for those who consider themselves more respectable and who wish to be more private. As you enter the larger of these rooms, a placard which is hung near the door informs you what the charges are, and a man stands there to receive the money on entrance. Arranged in rows down the middle and around the sides of both rooms, are a number of small boxes, or lockers, furnished with lock and key, into which the visitors put their clothes, and where they can make sure of them when they return from the bathing-room.

The bathing-room is entered by a small door at the further end of the building, and is about thirty feet long, and twenty wide ; the bath occupying the whole space, except a narrow path around the sides. The water is from one foot to eighteen inches deep, and the sides are lined and covered with marble slabs, from which the bathers step into the water, and on which they sit and wash themselves. The furnace is placed on the outside of one of the ends, and the flues are carried through below the centre of the bath. The establishment, in the afternoon and evening, is crowded with visitors, and, on entering the bath-room, the first impression is almost insupportable. The hot steam or vapor meets you at the door, filling the eyes and ears, and causing perspiration to run from every pore in the body ; it almost darkens the place, and the Chinamen seen in this imperfect light, with their brown skins and long tails, sporting in the water, render the scene a most ludicrous one to a foreigner. Those visitors who use the common room pay only six copper cash ; the other class pay eighteen—but they, in addition, have a cup of tea and a pipe of tobacco from the proprietors. I must mention that one hundred copper cash amount to about nine cents of our money, so that the first-class enjoy a hot water bath for about one-half a cent, and the other a bath, a private room, a cup of tea, and a pipe of tobacco for something less than two cents. From this it will be seen that the Chinese, although far behind us in many respects, could give our poorer classes a lesson in cleanliness.—F. M. in *Popular Science News.*

